# Physical activity and HIV in sub-Saharan Africa: a systematic review of correlates and levels

**DOI:** 10.4314/ahs.v18i2.25

**Published:** 2018-06

**Authors:** Davy Vancampfort, Brendon Stubbs, James Mugisha

**Affiliations:** 1 KU Leuven — University of Leuven, Department of Rehabilitation Sciences, Leuven, Belgium; 2 KU Leuven — University of Leuven, University Psychiatric Center KU Leuven, Leuven-Kortenberg, Belgium; 3 Physiotherapy Department, South London and Maudsley NHS Foundation Trust, London, UK; 4 Health Service and Population Research Department, Institute of Psychiatry, Psychology and Neuroscience, King's College London, De Crespigny Park, London, UK; 5 Butabika National Referral and Mental Health Hospital, Kampala, Uganda; 6 Kyambogo University, Kampala, Uganda

**Keywords:** Physical activity, exercise, physiotherapy, AIDS, HIV

## Abstract

**Background:**

Self-management strategies such as physical activity (PA) can address disability and optimize mental, physical, social and economic outcomes for persons living with HIV (PLWH). Understanding factors that influence PA behavior in PLWH is a first step in order to devise effective interventions.

**Objective:**

The present review provides a systematic review of the correlates of PA in PLWH in sub-Saharan Africa.

**Methods:**

Electronic databases were searched till April 2016. Keywords included ‘physical activity’ or ‘exercise’ or ‘sports’ and ‘AIDS’ or ‘HIV’.

**Results:**

Ten correlates were identified in 6 studies including 1,015 (329♂) PLWH (mean age range=30.5–40.8years). Lower levels of PA were associated with older age (2/2 studies), a lower number of CD4 cells/µl (1/1), a more severe HIV-stage (1/1), a higher HIV load (1/1), the presence of opportunistic infections (1/1) and a higher BMI (1/1). Fisher's exact tests showed there were more significant correlates in objective tools versus subjective self-report (P=0.03).

**Conclusion:**

The current review shows that participation in PA by PLWH in sub-Saharan Africa is associated with a range of complex factors which should be considered in the daily care of PLWH. This however might require repackaging of the current interventions for PLWH to allow a focus on PA.

## Introduction

Over the past decade, the landscape of HIV care in sub-Saharan Africa (SSA) has changed tremendously^1^. This is largely due to increased funding of HIV/AIDS care and research on the continent^2^. It has been reported that annual funding for AIDS in low and middle income countries increased 30-fold from 1996–2006, from US $ 300m to US $ 8.9 billion^2^. Due to increased funding, in many countries, testing and counselling services have been scaled up, while anti-retroviral therapy has become more widely available in the public health domain^3^. Despite this, many people living with HIV (PLWH) in SSA have poor long-term outcomes, and increased efforts through a multi-sectoral approach are urgently needed in order to reduce the HIV burden in this part of the world^4^. It has been shown that HIV leads to a decline in muscle function and reduced physical activity^5^, especially in women who are at the center of the economic production for the family in most African communities^6^. The effect of HIV/AIDS on the women has larger consequences because if their productivity is affected, this directly affects family welfare and increases the scale of both family and community poverty. In addition, in SSA, many HIV patients are relying on labor-demanding jobs in the informal sector with no job security or compensation for lost income. Maintaining physical strength and an adequate activity level is thus of crucial importance for their livelihoods^7^,^8^.

Self-management strategies, such as physical activity and exercise (as a structured form of physical activity) can address disability and optimize mental physical, social and economic outcomes in PLWH^9^–^14^. Similar benefits are observed in well-designed randomized controlled trials in SSA. For example, in a Nigerian study^15^ 45 to 60 min, 3 times/week for 8 weeks moderate intensity exercise (n=17) resulted in reduced blood pressure levels, improved aerobic fitness and increased CD4 cells versus conventional therapy involving anti-retroviral therapy and counseling only (n=16). Mangona et al.^16^ demonstrated in a community setting in Mozambique that a 13 weeks aerobic and resistance training program incorporating 20 minutes of cycling at moderate to high intensity and a muscular endurance circuit consisting of 6 exercises at 15 repetitions per minute resulted in improved cardiorespiratory fitness (n=19). Mutimura et al.^17^ on their turn showed that a 6-month supervised exercise program (3 times per week during 90 minutes) (n=50) at a fitness club in Kigali, Rwanda resulted in more social relationships, an improved quality of life, a better self-esteem and body image and less emotional stress than in the control group receiving care as usual.

Despite the observed benefits, a large proportion of PLWH in SSA are still not engaging in physical activity as part of their rehabilitation^18^ and drop-out rates from physical activity programs in SSA studies are up to 30%^15^–^17^. The major lacuna is the over-congested public health systems where almost all focus is put on delivering pharmacology (essential drugs) to patients with limited window to offer other public health packages^19^. Understanding cultural-specific barriers and facilitators of participation in physical activity in PLWH in SSA therefore is an essential first step in order to devise effective physical activity programs as conclusions drawn from physical activity interventions in PLWH in a Western society will not necessarily reflect those found in SSA^20^. Cultural-sensitive behavioral theories, such as the socio-ecological model^21^ have shown to be useful in attempting to understand the factors which influence physical activity behavior in vulnerable populations^22^–^26^. These models posit that multiple relevant attributes influence health behavior and include intrapersonal (demographic, biological, psychological, emotional and cognitive), interpersonal/cultural (e.g., social support, cultural habits), physical environment (e.g., distance to the facilities, financial costs, enjoyable scenery), and policy (laws, rules, regulations, codes) factors^21^. A few qualitative research studies conducted in South-African PLWH indicated that barriers to physical activity include physical complaints, e.g., low-energy levels, psychological complaints such as increased stress levels, family responsibility such as being primary caregivers, and the fear of disclosure and stigmatization, the physical environment including adverse weather conditions, the social environment including domestic abuse and crime, and the workplace situation, e.g., being in a sedentary job^27^,^28^. Facilitators of physical activity included support and encouragement from friends and family, religious practices during worship and community environment, e.g., having access to parks and sport fields^27^. In order to elaborate and confirm such qualitative findings, quantitative research in SSA-specific settings and which is able to identify potential correlates of actual physical activity levels in PLWH is needed. This information can then be used to target future physical activity interventions for PLWH in SSA. A systematic review on physical activity levels and correlates in PLWH in SSA is however currently lacking. Systematic quantitative research of physical activity levels and potential negative and positive correlates of physical activity in SSA will provide valuable information to implement physical activity in clinical settings and will inform future research. The present review therefore systematically evaluates published quantitative studies on physical activity levels and correlates in PLWH in SSA. In addition to summarizing methods and results of these studies, gaps in the current literature are identified and directions for future research in SSA contexts are proposed.

## Methods

This systematic review was conducted following an unpublished protocol and in accordance with the PRISMA guidelines^29^.

### Data sources and searches

Two independent reviewers (DV and BS) performed an electronic search of the health-related databases PubMed, CINAHL and Embase from database inception until April 1^st^ 2016. Manual searches were also conducted using the reference lists from identified articles. The medical subject headings used were ‘physical activity’ OR ‘exercise’ OR ‘sports’ AND ‘HIV’ OR ‘AIDS’ in the title, abstract or index term fields. We did not include a search term referring to SSA or any country in the region but instead checked all the retrieved articles manually.

### Eligibility criteria

Inclusion criteria were as follows: (a) a diagnosis of HIV or AIDS irrespective of the assessment method used, (b) participants were at least 18 years of age, (c) studies contained quantitative research and had been published in a peer-reviewed journal, (d) the dependent variable was a measure of physical activity participation; for the meta-analysis of the current levels the mean time (minutes) per day/week engaged in light, moderate or high intensity physical activity or sedentary behavior was needed, (e) the study was executed in a country of SSA. No restriction was placed on the selection of the outcome measure or the language of the article. For cohort or intervention studies, only associations of physical activity participation with baseline data were included. We excluded articles if the dependent variable was aerobic fitness, physical activity intention, self-efficacy, or other intermediate (non-behavioral) measures because these variables are less direct indicators of actual physical activity behavior^30^. Also, case reports and expert opinions were excluded.

### Data collection

Two reviewers (DV and BS) independently extracted data from the included studies using a pre-determined form. The form captured data in 6 domains including (a) country, (b) gender, (c) age (mean), (d) the quality of the physical activity measure, (e) physical activity correlates, and (f) the physical activity /sedentary levels. We collected separate levels for light, moderate and high intensity physical activity as defined by the original authors if this date were reported. The following categories were used to code the quality of the physical activity measure: (a) self-report, and (b) acceptable objective measurements for PLWH. Objective measurements included motion sensors such as accelerometers and pedometers. In accordance with previous physical activity correlates reviews^22^–^24^,^31^–^33^ the following potential physical activity correlate categories were included: (a) demographic, (b) biological, (c) psychological / cognitive / emotional, (d) behavioral attributes/skills, (e) social/cultural factors, (f) physical environment, and (g) policy factors. Variables were classified as ‘related’ or ‘not related’ to physical activity based on statistical significance, and the direction of association for related variables was identified. The detailed data tables were further analyzed to create tables that summarized the state of the literature on different variables.

### Coding associations with physical activity

A variety of statistical techniques were used to evaluate correlates, including uni-/ bivariate analyses, correlations, t-tests, and ANOVA. If both uni-/bivariate and multivariate tests were conducted, uni-/bivariate tests were reported for consistency across studies. The column ‘related to physical activity’ indicates, which studies reported significant associations between the variable and the physical activity measure. Direction of association is indicated with a ‘+’ or ‘−’. The column ‘unrelated to physical activity’ indicates which studies reported non-significant associations between the variable and physical activity.

### Summary codes

A summary code for each variable was presented and calculated following previous recommendations^34^,^35^. The summary code column contains a code to summarize the state of the literature for that variable. The percentages refer to the number of associations supporting the expected association divided by the total number of associations for the variable. Associations were coded with: ‘0’ (0–33% of studies supporting association); ‘?’ (34%–59% of studies supporting an association); or ‘+’ or ‘−’ (60%–100% of studies supporting an association).

### Differences in number of significant correlates

Using Fisher's exact tests, we explored differences in the number of significant correlates versus unrelated variables obtained via objective physical activity assessments versus subjective assessments.

## Results

### Study selection

**Figure 1:**
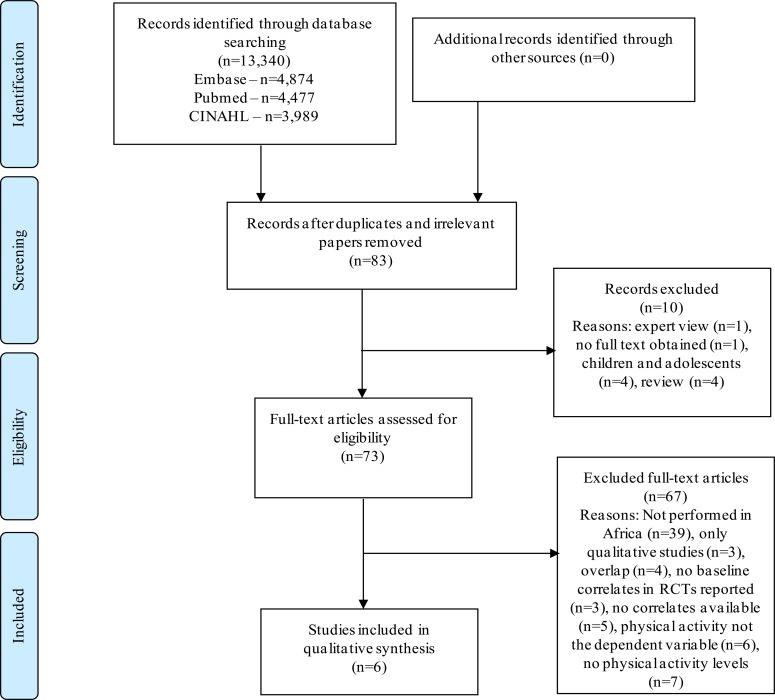
Flow diagram

Out of 83 potentially eligible studies, 6 were included in this review. The search strategy and reasons for exclusion are shown in Figure 1.

### Participant and study characteristics

Across all 6 studies^7^,^36^–^40^, a total of 1,015 (329♂) PLWH (mean age range=30.5–40.8years) were included in the analyses. The sample size ranged from 42 to 407. Three studies were executed in South-Africa, and one in Ethiopia, one in Malawi, and one in Nigeria. Concerning the quality of the physical activity measure, 4 studies were based on self-report measures and 2 studies used an objective measure of physical activity. Table 1 presents the characteristics of the included participants, the quality of physical activity assessments and the physical activity levels.

**Table 1:** Characteristics of the included physical activity studies

Nr	First author / year [Ref nr]	Country	Participants	PA measurement	Quality of PA Measurement	PA and sedentary behavior levels
1	Olsen 2015^7^	Ethiopia	116♂; mean age=37.6±8.6years; BMI=18.8±2.1; 232♀; mean age=30.5±7.8years; BMI=19.2±2.8	Accelerometer	B	♂ spent 75% of the time sedentary vs 77% in ♀; time spent in vigorous physical activity= 10% in ♂ and 5.2% in ♀
2	Roos 2014^36^	South-Africa	42 (7♂); mean age=38.7±8.9 years	Pedometer	B	8103±833 steps per day
3	Edward 2013^37^	Nigeria	265(86♂); mean age=38.7±8.7 years	STEP-wise approach to Surveillance	A	66% of the participants does not comply with general physical activity recommendations
4	Frantz 2013^38^	South-Africa	407 (93♂); mean age=38.8±8.9years	Sub-Saharan African Activity Questionnaire	A	62% (work-related) to 83% (leisure time related) of the participants does not comply with general physical activity recommendations
5	Muronya 2011^39^	Malawi	174 (67♂); mean age=40.8years	STEP-wise approach to Surveillance	A	68.2% reported never doing any vigorous exercise, and 12.7% never doing any moderate exercise. The mean duration of time spent resting per day was 9.9 hours, and 43.6% said they rested at least 12 hours per day.
6	Kinsey 2008^40^	South-Africa	186 (46♂ with a mean age=36±7years and 140♀ with a mean age=35±8years)	Combination MAQ, MLTPA and Baecke PAQ	A	♂: 770 ± 420 MET hours / month; ♀=869 ±443 MET hours/month

A=self-report of poor or unknown reliability/validity in persons living with HIV, B= objective PA assessment; MAQ=Modifiable Activity Questionnaire, MLTPA=Minnesota Leisure Time Physical Activity, PAQ=Physical Activity Questionnaire, MET=metabolic equivalent.

### Correlates of physical activity in PLWH in SSA

Table 2 summarizes associations between 10 potential correlates and the physical activity participation in PLWH.

**Table 2:** Summary of studies of correlates of physical activity in people living with HIV

Determinant variable	Significantly related to PA		Unrelated to PA	Summary code°	
	Study*	Assoc.	Study*	Assoc.	% studies reporting assoc.
***Demographic factors***					
Age (older)	38, 39	-		-	100% (2/2)
Gender (female)	7, 38	-	37, 39, 40	?	40% (2/5)
***Biological factors***					
Exposure to antiviral therapy (yes)	39	-	38, 40	0	33% (1/3)
Duration antiviral treatment (longer)			39	0	0% (0/1)
HIV status (WHO clinical stage higher)	7	-		-	100% (1/1)
HIV viral load (higher)	7	-		-	100% (1/1)
Number of CD4 cells/µl (lower)	7	-		-	100% (1/1)
Body mass index (higher)	7	-		-	100% (1/1)
Opportunistic infections (present)	7	-		-	100% (1/1)
***Behavioral attributes*** ***/skills***					
***Psychological, cognitive*** ***and emotional factors***					
***Social/cultural factors***					
***Physical environment***					
Food insecurity (present)	7M	+	7F	?	50% (1/2)
***Policy factors***					

PA=physical activity;

* Reference numbers;

° The percentages in parentheses refer to the number of associations supporting the expected association divided by the total number of associations for thevariable. Associations are coded with: “0” (0–33% of studies supporting association); “?” (34%–59% of studies supporting an association); or “+” or “−” (60%–100% of studies supporting an association).

### Demographic correlates

Older age (2/2 studies; 100%) was consistently associated with lower physical activity levels. Gender differences were inconsistently reported, i.e. 2 of 5 studies indicated women engaged in more PA than men, while 3 other studies showed no difference between genders.

### Biological correlates

Seven biological correlates were included. Exposure to anti-viral therapy (1/3, 33%) and duration of the anti-viral treatment (0/1, 0%) were unrelated to the level of physical activity. A lower number of CD4 cells/µl (1/1, 100%), a higher HIV stage (1/1, 100%), a higher HIV load (1/1, 100%), the presence of opportunistic infections (1/1, 100%) and a higher BMI (1/1, 100%) were negatively associated with physical activity.

### Behavioral attributes/skills

No behavioral attributes were examined.

### Psychological, cognitive and emotional correlates

No psychological, cognitive and emotional factors were examined.

### Social/cultural factors

No social and / or cultural factors were explored.

### Environmental factors

The presence of food insecurity was in Ethiopian men but not in women associated with more physical activity.

### Policy factors

No policy-level correlates were located in the systematic review of the literature.

### Differences in number of significant correlates

Fisher's exact tests showed there were more significant correlates in objective tools versus subjective self-report (P=0.0345).

## Discussion

### General findings and clinical implications

To the best of the authors' knowledge, the present review is the first to systematically document the correlates of physical activity in PLWH in SSA. Out of 10 correlates from 6 studies, we found that lower levels of physical activity were associated with older age^38^,^39^, a lower number of CD4 cells/µl^7^, a higher HIV stage^7^, a higher HIV load^7^, the presence of opportunistic infections^7^ and a higher BMI^7^. Of interest was that, more significant correlates were obtained in studies with an objective assessment. Although the available physical activity data for SSA settings are still limited, our varied findings clearly illustrate that participation in physical activity by PLWH in SSA is associated with a range of factors.

Knowledge about demographic correlates of physical activity behavior will help to identify these high-risk persons in whom physical activity is likely to be reduced and who may therefore require intensified and targeted interventions in the public sector. Such efforts are largely lacking in the current HIV/AIDS awareness campaigns in SSA. The current review shows that older patients with HIV are the most vulnerable patients^38^,^39^. This is not surprising since the majority of them in SSA are out of the productive sector, may live in social exclusion and face challenges related to their welfare. The observation that older age was associated with a lower physical activity participations is also in agreement with findings in the general population^35^. Our review points towards the need of considering in particular the lower levels of physical activity in elderly with HIV and this should be main streamed in programs that target the elderly population and specifically those that target the elderly with HIV/AIDS.

Government community development departments in SSA are normally in charge of the elderly and should work closely with public health departments and civil society organizations on this endeavor. A previous 12-month randomized study of an education and homebased pedometer walking program in 84 older PLWH in South-Africa^27^ demonstrated that a walking program improves physical activity levels, and reduces the presence and severity of cardiovascular risk factors. For example, compared with controls those in the walking intervention had better 6-minute walk test distance score (P=0.01), a more beneficial waist to hip ratio (P<0.001), lower glucose (P=0.001), and higher high-density lipoprotein (P= 0.01) levels over the 12-month period. More similar research is highly needed as it provides a starting point for thinking about the structural support needed by older persons with HIV, especially as in SSA, HIV erodes familial supports^8^. These studies should inform public health interventions on the alternative resources available for care for the elderly with HIV/AIDS in a context of weakening family systems. Focus could be put on the possibility to support and/or foster resilience of the traditional systems of care including the family and kinships.

The reason that a higher BMI was associated with less physical activity^7^ might be due to the fact that a higher BMI is also in SSA populations associated with more musculoskeletal problems^41^,^42^ and consequently might be associated with more bodily discomfort. Patients and health care professionals should however be informed that also in people with HIV in SSA physical activity enhances more bodily comfort, which in turn may benefit one's physical self-perceptions^17^,^28^. Future research should explore the prevalence of this problem in a SSA (context) and the techniques that can be employed to stimulate positive experiences and consequently support an enhanced sense of personal control over the body and it's functioning in PLWH. The current number of health workers (which is very low as compared to the patient load and limits health worker-patient interaction) should be addressed to allow health workers to interact with patients in a more comprehensive way including a focus on healthy lifestyle issues. Motivation of the few existing health workers to embrace physical activity could be a short term strategy in this direction since recruitment of health workers in SSA is a long process and resources may not be easily available in the short term^19^.

The fact that immunological parameters such as a lower number of CD4 cells/µl are associated with less physical activity^7^ might be due to the fact that this a measure for the disease severity and the need for treatment and might be associated with symptoms as nausea^43^, depression^44^ and bodily pain^45^. A systematic review of 61 studies in PLWH demonstrated that the prevalence of pain ranged from a point prevalence of 54% (95%CI=51%–56.%) to 83% (95%CI=76%–88%) using a three-month recall period^45^. The types of pain experienced by PLWH and the aetiology appear to vary. PLWH may experience pain as a direct result of the disease on peripheral or central nervous systems^45^. Pain may be due to resultant opportunistic infections as well, which was reported in this review as a negative correlate, or pain may arise as a result of the side effects of anti-retroviral treatment^46^. Exposure to and duration of antiviral treatment were however not associated with physical activity behavior. Despite an increasing awareness of pain as a significant contributor to the disability and impaired health related quality of life, the problem of its under-management persists^45^. Although there is preliminary evidence^47^,^48^ that inclusion of physiotherapy as a complementary treatment for pain-management and simultaneously improving mental and physical health outcomes and reducing disability is promising in resource-limited areas of SSA^49^. The need for regular and comprehensive assessment of patients including their mental health is recommended here. This might however require re-tooling of the existing health staff in terms of skills and other competencies to provide comprehensive care. The long term strategy is to focus on the public health training at medical institutions and other relevant institutions in SSA and build their capacity in training cadres that can provide comprehensive care.

Finally, one study^7^ showed that higher HIV loads were associated with less physical activity participation. Although a high HIV load should not be a contra-indication for physical activity, it is recommended that patients with high HIV viral load levels perform moderate instead of high intensity physical activity. While moderate intensity physical activity improves the immune function in PLWH^15^,^50^, high-intensity exercise is known to have immunosuppressive effects in PLWH^51^. Effective management of HIV and support to those infected is vital in increasing the opportunity to undertake physical activity. Issues of compliance and potential adverse events are also vital and should be addressed; though we don't have information pertaining to this in the current review.

### Limitations and recommendations for future research

There are several limitations to this review, which should be acknowledged. First, there is a large diversity with regard to social and community support, access to resources, family structures, living conditions, and HIV-related perceptions and stigma between the different SSA countries which should be acknowledged. Second, the diversity of physical activity measures poses a challenge to interpret the current findings. Self-report questionnaires are known to require motivation to complete all of the questions and often the detail regarding the level (frequency, duration and intensity) and type of physical activity is not consistently evaluated by questionnaires. To the best of our knowledge only the IPAQ^52^ and the Baecke physical activity questionnaire^53^ have been validated in PLWH so far. However the current review shows that fewer significant associations were found in self-report measures. Considering the wide diversity in physical activity assessments, our findings do reveal that there is a high need for researchers to adopt a clear consensus on which assessment tools should be recommended in PLWH. Although objective assessment should be preferred, they are often too costly to be used in resource-limited settings. Nevertheless, policy makers are highly recommended to invest in these tools as they are easy to use, particularly in more illiterate populations.

Third, all correlates investigated were only documented in a one or two studies. Examination of the same, standardized variables in more studies therefore is necessary in order to build a consistent body of evidence that can support or refute the potential influence of individual variables. Future research should focus on psychological, social, environmental and policy related factors associated with physical activity participation in SSA. For example, previous qualitative research indicated that PLWH in South-Africa living in a low socio-economic, disadvantaged settings have personal motives to be physically active, e.g., improving specific health parameters and a healthy and fit rather than a thin body appearance. In contrast with the Western ideal of a thin body shape, fear of HIV-related stigma encourage black African women to be rather slightly overweight, but not obese, than thin and having people think they were infected with HIV or they had AIDS^54^. One model that might be useful to test individual motives in a more rigorous way and which recently has been adopted successfully in a physical activity study in American PLWH^55^ is the self-determination theory (SDT)^56^. The theory proposes motivation towards physical activity is multi-dimensional and resides along a continuum.

The lowest end of the continuum is identified as a motivation which represents a general lack of motivation to change behaviour due to discouragement. Following along the continuum, external regulation refers to being physically active or exercising to avoid criticism or to obtain promised rewards or external appreciation. Introjected regulation refers to the imposition of pressures onto one's own functioning, for instance, by reinforcing one's activity engagement with feelings of guilt, self-criticism, or contingent self-worth. More volitional (or autonomous) forms of functioning include identified regulation, which involves foreseeing the personal importance of physical activity, and integrated regulation, which implies that physical activity is brought in harmony with other prevailing life values, such that being active becomes prioritised within one's healthy lifestyle. Finally, intrinsic motivation involves engaging in physical activity for its own sake, that is, because one finds being active stimulating or enjoyable by itself. Qualified professionals should facilitate PLWH in becoming self-determined (autonomous) to engage in activities, while at the same time internalizing the motivation to regulated behaviors, which may not be initially interesting or valued. To foster this internalization, a patient-centered approach is needed. An approach that satisfies the psychological needs of relatedness, autonomy, and competence is encouraged^56^. Longitudinal and intervention studies in SSA settings are needed in order to test the SDT-principles in SSA. These studies should however also consider the notion of collectivism in which the needs of the group are prioritised above one's own needs within the group. From this collective perspective people should not engage in any activities that would benefit only the individual before everyone in the group is taken care off.

Previous qualitative research in South-Africa^27^ indicated that family and friends are significant facilitators of physical activity in PLWH. They can for example provide support and encouragement by sporadically walking with them and encouraging participants to walk when sitting too much. More research is however needed on the amount and type of social support necessary to begin and maintain physical activity behavior in PLWH in SSA. This kind of research could explore whether: (a) the relationship between physical activity participation and social support is a dynamic process in which the sources of support or need for support change over time, and (b) any social barriers for PLWH can be identified and addressed by involving significant others.

Next, environmental modifications such as making physical activity facilities easily accessible should be evaluated. Having access to parks or sport fields facilitates walking. Additionally, in rural communities, as long as their health status allows it, PLWH have to go for long distances to fetch water and wood^27^. There are self-report tools available for the role of the physical environment on a person's physical activity behavior in disadvantaged settings in SSA^57^. These tools should be applied in PLWH.

Correlates at policy level may probably be best initially explored using a qualitative approach^31^. Now that the SSA has quite a high level of funding to the HIV/AIDS sector, public health actors need to mainstream physical activity in HIV/AIDS interventions. The focus on strengthening primary health care systems in SSA Africa should augment such concerns/interest in physical activity being part of HIV/AIDS programs. Researchers should examine, which policies are currently in place to motivate PLWH in specific SSA countries to an active and healthy lifestyle. Interviews of PLWH but also health care professionals and policy makers may provide further insight as to what is needed to stimulate an active lifestyle in these countries. If the purpose is to inform and motivate policy changes that will improve the quality of life and reduce the disability in PLWH merely documenting the relationship between policy initiatives but also for example environmental changes and physical activity behavior is likely to be insufficient. At some point, environmental and policy change research will need to include assessments of broader health outcomes in PLWH, such as changes in the prevalence of chronic co-morbidities, health care service utilization, as well as the economic costs and benefits of proposed policy changes.

## Conclusion

The current review shows that participation in PA by PLWH in SSA is associated with a range of demographical and biological factors which should be considered in the daily care of PLWH. More research is highly needed in order to define psychological, social, environmental and policy related factors that might influence PA behavior in PLWH in this part of the world. Policy directions were discussed in this review.

## References

[1] World Health Organization (2013). Global update on HIV treatment 2013: results, impact and opportunities.

[2] Oomman N, Bernstein M, Rosenzweig S (2007). Following the funding for HIV/AIDS: a comparative analysis of the funding practices of PEPFAR the Global Fund and World Bank MAP in Mozambique Uganda and Zambia.

[3] Baggaley R, Hensen B, Ajose O, Grabbe KL, Wong VJ, Schilsky A (2012). From caution to urgency: the evolution of HIV testing and counselling in Africa. Bulletin of the World Health Organization.

[4] Atun R, Chang AY, Ogbuoji O, Silva S, Resch S, Hontelez J (2016). Long-term financing needs for HIV control in sub-Saharan Africa in 2015–2050: PubMed a modelling study. BMJ Open.

[5] Macallan DC, Noble C, Baldwin C, Jebb SA, Prentice AM, Coward WA (1995). Energy expenditure and wasting in human immunodeficiency virus infection. New England Journal of Medicine.

[6] Pathai S, Gilbert C, Weiss HA, Cook C, Wood R, Bekker L-G (2013). Frailty in HIV-infected adults in South Africa. Journal of Acquired Immune Deficiency Syndromes (1999).

[7] Olsen MF, Kæstel P, Tesfaye M, Abdissa A, Yilma D, Girma T (2015). Physical activity and capacity at initiation of antiretroviral treatment in HIV patients in Ethiopia. Epidemiology and Infection.

[8] Schatz E, Gilbert L (2014). “My legs affect me a lot.… I can no longer walk to the forest to fetch firewood”: Challenges related to health and the performance of daily tasks for older women in a high HIV context. Health Care for Women International.

[9] Leach L, Bassett S, Smithdorf G, Andrews B, Travill A (2015). A systematic review of the effects of exercise interventions on body composition in HIV+ adults. The Open AIDS Journal.

[10] Gomes-Neto M, Conceição CS, Carvalho VO, Brites C (2015). Effects of combined aerobic and resistance exercise on exercise capacity, muscle strength and quality of life in HIV-infected patients: a systematic review and meta-analysis. PloS One.

[11] Gomes-Neto M, Conceicao CS, Carvalho VO, Brites C (2013). A systematic review of the effects of different types of therapeutic exercise on physiologic and functional measurements in patients with HIV/AIDS. Clinics.

[12] Gomes Neto M, Ogalha C, Andrade AM, Brites C (2013). A systematic review of effects of concurrent strength and endurance training on the health-related quality of life and cardiopulmonary status in patients with HIV/AIDS. BioMed Research International.

[13] Fillipas S, Cherry C, Cicuttini F, Smirneos L, Holland A (2010). The effects of exercise training on metabolic and morphological outcomes for people living with HIV: a systematic review of randomised controlled trials. HIV Clinical Trials.

[14] O'Brien K, Tynan A-M, Nixon S, Glazier R (2008). Effects of progressive resistive exercise in adults living with HIV/AIDS: systematic review and meta-analysis of randomized trials. AIDS Care.

[15] Ezema C, Onwunali A, Lamina S, Ezugwu U, Amaeze A, Nwankwo M (2014). Effect of aerobic exercise training on cardiovascular parameters and CD4 cell count of people living with human immunodeficiency virus/acquired immune deficiency syndrome: A randomized controlled trial. Nigerian Journal of Clinical Practice.

[16] Mangona L, Daca T, Tchonga F, Bule O, Bhatt N, Jani I (2015). Effect of different types of exercise in HIV+ Mozambican women using antiretroviral therapy. The Open AIDS Journal.

[17] Mutimura E, Stewart A, Crowther NJ, Yarasheski KE, Cade WT (2008). The effects of exercise training on quality of life in HAART-treated HIV-positive Rwandan subjects with body fat redistribution. Quality of Life Research.

[18] Schuelter-Trevisol F, H Wolff F, R Alencastro P, Grigoletti S, L Ikeda M, Brandao A (2012). Physical activity: do patients infected with HIV practice? How much? A systematic review. Current HIV Research.

[19] Mugisha J, Ssebunnya J, Kigozi FN (2016). Towards understanding governance issues in integration of mental health into primary health care in Uganda. International Journal of Mental Health Systems.

[20] Ley C, Prista A (2015). Physical activity and HIV in Africa. The Open AIDS Journal.

[21] Sallis JF, Cervero RB, Ascher W, Henderson KA, Kraft MK, Kerr J (2006). An ecological approach to creating active living communities. Annu Rev Public Health.

[22] Stubbs B, Eggermont L, Soundy A, Probst M, Vandenbulcke M, Vancampfort D (2014). What are the factors associated with physical activity (PA) participation in community dwelling adults with dementia? A systematic review of PA correlates. Archives of Gerontology and Geriatrics.

[23] Stubbs B, Hurley M, Smith T (2015). What are the factors that influence physical activity participation in adults with knee and hip osteoarthritis? A systematic review of physical activity correlates. Clinical rehabilitation.

[24] Vancampfort D, Vanderlinden J, Stubbs B, Soundy A, Pieters G, Hert MD (2014). Physical activity correlates in persons with binge eating disorder: A systematic review. European Eating Disorders Review.

[25] Vancampfort D, Knapen J, Probst M, Scheewe T, Remans S, De Hert M (2012). A systematic review of correlates of physical activity in patients with schizophrenia. Acta Psychiatrica Scandinavica.

[26] Vancampfort D, De Hert M, Stubbs B, Soundy A, De Herdt A, Detraux J (2015). A systematic review of physical activity correlates in alcohol use disorders. Archives of Psychiatric Nursing.

[27] Roos R, Myezwa H, van Aswegen H (2015). “Not easy at all but I am trying”: barriers and facilitators to physical activity in a South African cohort ofpeople living with HIV participating in a home-based pedometer walking programme. AIDS Care.

[28] Ley C, Barrio MR, Leach L (2015). Social-ecological, motivational and volitional factors for initiating and maintaining physical activity in the context of HIV. The Open AIDS Journal.

[29] Moher D, Liberati A, Tetzlaff J, Altman DG (2009). Preferred Reporting Items for Systematic Reviews and Meta-Analyses: The PRISMA Statement. PLoS Med.

[30] Caspersen CJ, Powell KE, Christenson GM (1985). Physical activity, exercise, and physical fitness: definitions and distinctions for health-related research. Public Health Reports.

[31] Vancampfort D, Correll CU, Probst M, Sienaert P, Wyckaert S, De Herdt A (2013). A review of physical activity correlates in patients with bipolar disorder. Journal of Affective Disorders.

[32] Vancampfort D, De Hert M, Stubbs B, Soundy A, De Herdt A, Detraux J (2015). A systematic review of physical activity correlates in alcohol use disorders. Archives of Psychiatric Nursing.

[33] Vancampfort D, Stubbs B, Sienaert P, Wyckaert S, De Hert M, Rosenbaum S (2015). What are the factors that influence physical activity participation in individuals with depression? A review of physical activity correlates from 59 studies. Psychiatria Danubina.

[34] Sallis JF, Prochaska JJ, Taylor WC (2000). A review of correlates of physical activity of children and adolescents. Medicine and Science in Sports and Exercise.

[35] Trost SG, Owen N, Bauman AE, Sallis JF, Brown W (2002). Correlates of adults' participation in physical activity: review and update. Medicine and Science in Sports and Exercise.

[36] Roos R, Myezwa H, van Aswegen H, Musenge E (2014). Effects of an education and home-based pedometer walking program on ischemic heart disease risk factors in people infected with HIV: a randomized trial. JAIDS Journal of Acquired Immune Deficiency Syndromes.

[37] Edward AO, Oladayo AA, Omolola AS, Adetiloye AA, Adedayo PA (2013). Prevalence of traditional cardiovascular risk factors and evaluation of cardiovascular risk using three risk equations in Nigerians living with human immunodeficiency virus. North American Journal of Medical Sciences.

[38] Frantz JM, Murenzi A (2013). The physical activity levels among people living with human immunodeficiency virus/acquired immunodeficiency syndrome receiving high active antiretroviral therapy in Rwanda. Sahara Journal.

[39] Muronya W, Sanga E, Talama G, Kumwenda JJ, van Oosterhout JJ (2011). Cardiovascular risk factors in adult Malawians on long-term antiretroviral therapy. Transactions of the Royal Society of Tropical Medicine and Hygiene.

[40] Kinsey K, McVeigh J, Chantler I (2008). Habitual physical activity levels are positively correlated with CD4 counts in an HIV-positive South African population. African Journal of AIDS Research.

[41] Koyanagi A, Stickley A, Garin N, Miret M, Ayuso-Mateos JL, Leonardi M (2015). The association between obesity and back pain in nine countries: a cross-sectional study. BMC Public Health.

[42] Fabunmi A, Aba S, Odunaiya N (2005). Prevalence of low back pain among peasant farmers in a rural community in South West Nigeria. African Journal of Medicine and Medical Sciences.

[43] Chubineh S, McGowan J (2008). Nausea and vomiting in HIV: a symptom review. International Journal of STD & AIDS.

[44] Pala AN, Steca P, Bagrodia R, Helpman L, Colangeli V, Viale P (2016). Subtypes of depressive symptoms and inflammatory biomarkers: An exploratory study on a sample of HIV-positive patients. Brain, Behavior, and Immunity.

[45] Parker R, Stein DJ, Jelsma J (2014). Pain in people living with HIV/AIDS: a systematic review. Journal of the International AIDS Society.

[46] Hewitt DJ, McDonald M, Portenoy RK, Rosenfeld B, Passik S, Breitbart W (1997). Pain syndromes and etiologies in ambulatory AIDS patients. Pain.

[47] Pullen SD, Chigbo NN, Nwigwe EC, Chukwuka CJ, Amah CC, Idu SC (2014). Physiotherapy intervention as a complementary treatment for people living with HIV/AIDS. HIV/AIDS (Auckland, NZ).

[48] Mgbemena O, Westfall AO, Ritchie CS, Hicks J, Raper JL, Overton ET (2015). Preliminary outcomes of a pilot physical therapy program for HIV-infected patients with chronic pain. AIDS Care.

[49] Mkandla K, Myezwa H, Musenge E (2015). The effects of progressive-resisted exercises on muscle strength and health-related quality of life in persons withHIV-related poly-neuropathy in Zimbabwe. AIDS Care.

[50] Garcia A, Fraga GA, Vieira RC, Silva CMS, Trombeta JCDS, Navalta JW (2014). Effects of combined exercise training on immunological, physical and biochemical parameters in individuals with HIV/AIDS. Journal of Sports Sciences.

[51] d'Ettorre G, Ceccarelli G, Giustini N, Mastroianni CM, Silvestri G, Vullo V (2014). Taming HIV-related inflammation with physical activity: a matter of timing. AIDS research and Human Retroviruses.

[52] Fillipas S, Cicuttini F, Holland AE, Cherry CL (2010). The international physical activity questionnaire overestimates moderate and vigorous physical activity in HIV-infected individuals compared with accelerometry. Journal of the Association of Nurses in AIDS Care.

[53] Florindo AA, Latorre MdRDd, Santos ECMd, Negrão CE, Azevedo LF, Segurado AAC (2006). Validity and reliability of the Baecke questionnaire for the evaluation of habitual physical activity among people living with HIV/AIDS. Cadernos de Saúde Pública.

[54] Matoti-Mvalo T, Puoane T (2011). Perceptions of body size and its association with HIV/AIDS. South African Journal of Clinical Nutrition.

[55] Shah KN, Majeed Z, Yoruk YB, Yang H, Hilton TN, McMahon JM (2016). Enhancing physical function in HIV-infected older adults: a randomized controlled clinical trial.

[56] Deci EL, Ryan RM (2000). The” what” and” why” of goal pursuits: Human needs and the self-determination of behavior. Psychological inquiry.

[57] Oyeyemi AL, Kasoma SS, Onywera VO, Assah F, Adedoyin RA, Conway TL (2016). NEWS for Africa: adaptation and reliability of a built environment questionnaire for physical activity in seven African countries. International Journal of Behavioral Nutrition and Physical Activity.

